# Profil épidémiologique et prise en charge de l’éclampsie au Sénégal: à propos de 62 cas

**DOI:** 10.11604/pamj.2013.16.83.3101

**Published:** 2013-11-06

**Authors:** Abdoul Aziz Diouf, Moussa Diallo, Magatte Mbaye, Sokhna Diarra Sarr, Marie Edouard Faye-Diémé, Jean Charles Moreau, Alassane Diouf

**Affiliations:** 1 UCAD, Clinique Gynécologique et Obstétricale, Dakar, Sénégal

**Keywords:** Eclampsie, prééclampsie, sulfate de magnésium, Sénégal, eclampsia, preeclampsia, magnesium sulfate, Senegal

## Abstract

**Introduction:**

L'objectif de cette étude etait d'apprécier les caractéristiques épidémiologiques et cliniques de l’éclampsie et d’évaluer la prise en charge et le pronostic maternel et périnatal.

**Méthodes:**

Étude descriptive rétrospective sur 3 années (2007-2010) dans un service de Gynécologie Obstétrique de Dakar (Sénégal). Le critère d'inclusion était toute crise convulsive en période gravido-puerpérale dans un contexte de prééclampsie.

**Résultats:**

Sur un total de 4587 accouchements, 62 cas d’éclampsie étaient enregistrés représentant une incidence de 1,35%. Le profil retrouvé était celui d'une jeune femme (24 ans), primipare (58.1%), habitant la banlieue dakaroise (83.8%), porteuse d'une grossesse à terme (56.5%), mal suivie (82.3%) et référée par un poste de santé environnant (82.3%). La crise était survenue en antépartum et en post-partum dans 72.5 et 27,5% respectivement. Toutes les patientes présentaient une HTA ; l’‘dème et la protéinurie étaient retrouvés dans 72.5 et 84% respectivement. La majorité des patientes (88%) avait présenté plus de deux crises et l’état de mal éclamptique concernait 14.5% des cas. Le sulfate de magnésium était utilisé chez toutes les patientes. La césarienne était le mode d'accouchement largement adopté (75.5%) pour les patientes reçues en antépartum. Le pronostic maternel était marqué par un cas de décès. La mortalité périnatale était de 130%.

**Conclusion:**

L’éclampsie est un problème de santé publique dans les pays en développement. Les principaux facteurs de risque sont la primiparité et l’âge jeûne. L'administration du sulfate de magnésium et la césarienne permettent d'améliorer le pronostic maternel et foetal. La prévention passe nécessairement par un suivi prénatal de qualité.

## Introduction

Avec plus de 50.000 décès dans le monde [1], l’éclampsie garde une place prépondérante dans les causes de mortalité maternelle. Si elle est devenue rare dans les pays développés, elle reste toujours présente en Afrique subsaharienne où le suivi prénatal de qualité fait encore défaut. Sa prise en charge est bien codifiée depuis l'utilisation du sulfate de magnésium dans ses protocoles thérapeutiques ; toutefois, la morbidité et la mortalité maternelle et surtout périnatale par éclampsie restent toujours élevées. Il importe ainsi de se concentrer sur la prévention qui doit passer par une meilleure connaissance épidémiologique de cette complication paroxystique de la prééclampsie. L'objectif de notre étude était de revoir les particularités épidémiologiques de l’éclampsie pouvant justifier sa fréquence constante, d’évaluer la prise en charge dans notre structure et d'apprécier le pronostic maternel et périnatal.

## Méthodes

Il s'agissait d'une étude rétrospective, descriptive allant du 1^er^ janvier 2007 au 1^er^ janvier 2010. Etaient incluse toute femme reçue au service des urgences de notre structure sanitaire pour des convulsions généralisées et/ou de troubles de la conscience, survenant entre la 20^ème^ Semaine d'Aménorrhée (SA) et la 6^ème^ semaine du post-partum sur un terrain d'hypertension artérielle (HTA). Pour éviter les biais de sélection, le diagnostic devait être systématiquement confirmé par un gynécologue. Etaient exclues de l’étude toutes les patientes qui présentaient une crise convulsive en l'absence d'HTA et ou de protéinurie. Soixante-deux dossiers de patientes reçues aux urgences obstétricales pour crise d’éclampsie ont été colligés.

Les sources des données étaient constituées par les carnets de consultation prénatale, les dossiers d'accouchement, et les dossiers d'hospitalisation. Les variables étudiées étaient les caractéristiques socio-démographiques, le déroulement de la grossesse, de l'accouchement et les paramètres néonatals. Les données étaient saisies et analysées grâce au logiciel SPSS 11.0. En cas de mesure d'association épidémiologique positive, l'intervalle de confiance (IC) à 95% était calculé selon la méthode de Miettinen. La signification de l'association était vérifiée en utilisant le test de Chi 2 de Pearson avec une valeur de p inférieure à 0.05.

## Résultats

**Figure 1:**
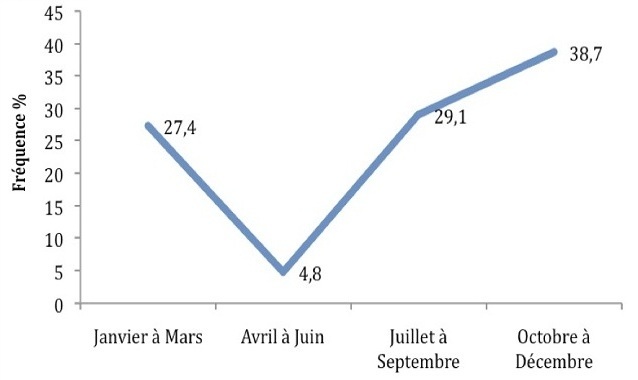
Fréquence des crises d’éclampsie en fonction des mois de l'année

Le nombre d'accouchement durant cette période était de 4587. La prévalence de l’éclampsie était de 1.35%. Les caractéristiques générales des patientes sont détaillées au Tableau 1. Le profil épidémiologique était celui d'une primipare (58.1%) âgée en moyenne de 24 ans, porteuse d'une grossesse d’âge gestationnel supérieur à 37 SA (56.5%), mal suivie (82.3%) et référée d'urgence par un poste de santé environnant (82.3%). La crise était survenue dans 45% des cas pendant la grossesse, dans 27% et 28% des cas respectivement pendant l'accouchement et le post-partum. Les crises convulsives les plus tardives étaient survenues chez une patiente 5 jours après un accouchement à domicile. Concernant la période de survenue (Figure 1), la fréquence de l’éclampsie était de 4,8% au deuxième trimestre de l'année (Avril-Mai-Juin), alors qu'elle était de 38.7% au dernier trimestre de l'année (Octobre-Novembre-Décembre). Toutes les patientes présentaient une hypertension artérielle; l'oedème et la protéinurie étaient retrouvés dans 72.5% et 84% respectivement. La majorité des patientes (88%) avait présenté plus de deux crises et l’état de mal éclamptique concernait 14.5% des cas. Le nombre d’épisodes de crise n'avait aucune influence significative sur la morbidité maternelle et néonatale (p = 0.02). Le traitement antihypertenseur à base de nicardipine était administré dans 88% des cas alors que le sulfate de magnésium en intraveineuse selon le protocole de l'OMS était utilisé chez toutes les patientes. Aucune récidive de crise n'a été observée après administration du traitement d'attaque au sulfate de magnésium. La césarienne était le mode d'accouchement largement adopté (75.5%) pour les patientes reçues en antépartum. Le pronostic maternel était marqué par un cas de décès par coagulation intra-vasculaire disséminée et la survenue de complications telles que le syndrome HELLP (8%), l'insuffisance rénale aigue (8%) et l'hématome rétroplacentaire (6.5%). Le retentissement foetal était plus important avec 9 cas de prématurité et 7 cas de mort foetale in utero. La mortalité périnatale était de 130 per 1000 elle était significativement associée à un âge gestationnel inférieur 37 SA (p = 0.008) et l'existence d'une tension artérielle diastolique supérieure ou égale à 110 mm Hg (p = 0.03) Tableau 2.

**Table 1:** Caractéristiques générales de la population d’étude

	Nombre	Pourcentage
**Age**		
15 – 25 ans	30	48.4
26 – 34 ans	28	45.2
≥ 35 ans	4	6.4
**Parité**		
Primipares	36	58.1
Paucipares	12	19.4
Multipares	14	22.5
**Terme de grossesse**		
≤ 34 SA	10	16.1
34 – 36 SA	15	24.2
≥ 37 SA	37	59.7
**Nombre de crises**		
Une crise	2	3.2
Deux crises	21	33.9
≥ 3 crises	39	62.9
**Moment de survenue**		
Antépartum	28	45
Per-partum	17	27.5
Post-partum	17	27.5
**Voie d'accouchement**		
Voie basse	28	45.2
Césarienne	34	54.8

**Table 2:** Complications maternelles et périnatales par éclampsie

	Nombre	Pourcentage
**Complications maternelles**		
HELLP syndrome	5	8%
Insuffisance rénale aigue	5	8%
Hématome rétroplacentaire	4	6.5%
**Complications périnatales**		
Accouchements prématurés	9	14.5
Retard de croissance intra-utérin	2	3.2
Asphyxie fœtale	2	3.2
Mort fœtale in utero	7	11.4

## Discussion

L’éclampsie est devenue une complication rare dans les pays développés grâce une prise en charge précoce devant l'apparition d'un des principaux signes de prééclampsie. D'autre part, la surveillance des grossesses par un personnel de santé qualifié, le dépistage des grossesses à risque et l'information des patientes ont permis de faire régresser cette pathologie [2]. Toutefois, elle reste fréquente dans nos pays en développement (1.35% dans notre série) et cette prévalence est presque constante en Afrique Subsaharienne [3–5]. Ce taux élevé est lié à plusieurs facteurs qui ont comme dénominateur commun le bas niveau socio-économique des populations. Dans notre série, le problème majeur retrouvé était la mauvaise prise en charge des états hypertensifs, en effet, les insuffisances notées dans le suivi prénatal ne permettaient pas de dépister à temps les HTA gravidiques.

A l’échelle nationale, ces chiffres devront être revus à la hausse, car la plupart des études ne permettent de recenser que les cas reçus dans les structures de référence. Ce qui signifie que la prévalence dans les zones rurales ou inaccessibles est mal appréciée.

Par ailleurs, nous avons retrouvé dans notre série une certaine variation de la fréquence de l’éclampsie en fonction des différentes périodes de l'année, avec un pic en période de froid. Cet aspect a été rapporté dans les études de Dao et al. [5] et d'Elongi et al. [6] qui suggéraient qu'il y'aurait une association entre humidité, basses températures et risque de survenue d’éclampsie.

Habituellement, la crise survient dans le dernier tiers de la grossesse plus que dans le post-partum comme en atteste notre étude ainsi que des données de la littérature [2, 7–9]. Cependant, du fait de l'absence de grossesse, l’éclampsie du post-partum est globalement de meilleur pronostic comparée aux autres moments de survenue.

L’évolution de la maladie gravidique de la prééclampsie vers l’éclampsie ne se discute plus. En effet, toutes les patientes de notre série avaient présenté une HTA gravidique ou une prééclampsie avant la survenue de la crise. Au vu de nos résultats, il semble que l'existence de crises inaugurales relatées par certains auteurs [10] ne relève que d'un défaut de dépistage des éléments du syndrome de la prééclampsie. Cependant, il est clair que tous les critères de la maladie gravidique ne sont pas indispensables à la survenue d'une crise, surtout que les signes annonciateurs peuvent être difficiles à interpréter du fait de leur caractère atypique. Dans tous les cas, il est légitime de conclure que la recherche systématique des prodromes et l'utilisation préventive du sulfate de magnésium pourraient permettre d’éviter l’éclampsie.

Plutôt que le nombre d’épisode de convulsion, c'est l'absence de prise en charge précoce qui constitue un facteur de mauvais pronostic maternel et foetal, car aucune récidive de crise n'a été observée après institution du protocole de sulfate de magnésium quel que soit le nombre de convulsions antérieures.

L’éclampsie est l’étape ultime de la maladie gravidique. Il s'agit d'un accident systémique qui s'accompagne le plus souvent d'un cortège de complications, rendant le pronostic encore plus défavorable. Le syndrome HELLP (8%), l'insuffisance rénale aigue (8%), l'hématome rétroplacentaire (6%) sont souvent retrouvés par d'autres auteurs ; leur fréquence parait être élevée en antépartum [11, 12].

La voie d'accouchement privilégiée en dehors du travail reste pour beaucoup d'auteurs la césarienne [13] comme il a été le cas dans notre série. L'anesthésie utilisée est générale même en cas de réveil temporaire avec une conscience normale. En dehors de l’évacuation utérine indiscutable et sans délai, la prise en charge médicamenteuse repose sur l'administration de sulfate de magnésium selon l'OMS, comme il a été le cas dans notre étude pour le traitement et la prévention des convulsions. Son utilisation s'est en outre révélée efficace dans la prévention des crises comme en atteste notre travail, vu l'absence des récidives, d'une part et d'autre part chez les patientes présentant une prééclampsie sévère avec des signes neurologiques [14]. Ce traitement peut être associé au Diazépam dans l'urgence.

Si nous comparons notre létalité (1,62%) par rapport aux autres séries africaines telles que celles Dao et al. (15%), ou Moussaoui et al. (2,4%) [5, 15] nous pouvons estimer que nous avons un taux très acceptable. Ce résultat émane d'une collaboration exemplaire entre les services d'obstétrique et de réanimation. Cependant, l'objectif ultime reste une mortalité maternelle nulle comme dans la série de Ducarme en France [2].

L'issue foetale était moins favorable que celle maternelle, avec une mortalité périnatale de 130.4%, liée essentiellement à la prématurité. Pour d'autres auteurs, ce taux est variable, dépendant probablement du contexte de prise en charge néonatale. Ainsi, dans la série de Bèye [3], la mortalité périnatale était de 428%, Mayi-tsonga et al. [14] trouvaient 170%, et Ducarme et al. [2] 125%.

## Conclusion

L’éclampsie reste toujours fréquente dans nos régions. Elle survient préférentiellement chez la jeune primipare présentant une HTA et ou une protéinurie sur une grossesse mal suivie. Le sulfate de magnésium et la réalisation de la césarienne permettent d'améliorer le pronostic maternel et foetal. La prévention passe nécessairement par un suivi prénatal de qualité.
